# Baseline-adjusted proportional odds models for the quantification of treatment effects in trials with ordinal sum score outcomes

**DOI:** 10.1186/s12874-020-00984-2

**Published:** 2020-05-06

**Authors:** Muriel Buri, Armin Curt, John Steeves, Torsten Hothorn

**Affiliations:** 1 Department of Biostatistics, Epidemiology, Biostatistics and Prevention Institute, University of Zurich, Hirschengraben 84, Zurich, CH-8001 Switzerland; 2 University Hospital Balgrist, Spinal Cord Injury Center, Forchstrasse 340, Zurich, CH-8008 Switzerland; 3 International Collaboration On Repair Discoveries (ICORD), University of British Columbia, Vancouver/Kelowna, Canada

**Keywords:** Transformation model, Distribution regression, Ordinal scores, Odds ratio, Spinal cord, EMSCI, NISCI, Sygen®, Clinical trial, Upper extremity motor score (UEMS), Spinal cord independence measure (SCIM)

## Abstract

**Background:**

Sum scores of ordinal outcomes are common in randomized clinical trials. The approaches routinely employed for assessing treatment effects, such as *t*-tests or Wilcoxon tests, are not particularly powerful in detecting changes in relevant parameters or lack the ability to incorporate baseline information. Hence, tailored statistical methods are needed for the analysis of ordinal outcomes in clinical research.

**Methods:**

We propose baseline-adjusted proportional odds logistic regression models to overcome previous limitations in the analysis of ordinal outcomes in randomized clinical trials. For the validation of our method, we focus on common ordinal sum score outcomes of neurological clinical trials such as the upper extremity motor score, the spinal cord independence measure, and the self-care subscore of the latter. We compare the statistical power of our models to other conventional approaches in a large simulation study of two-arm randomized clinical trials based on data from the European Multicenter Study about Spinal Cord Injury (EMSCI, ClinicalTrials.gov Identifier: NCT01571531). We also use the new method as an alternative analysis of the historical Sygen®clinical trial.

**Results:**

The simulation study of all postulated trial settings demonstrated that the statistical power of the novel method was greater than that of conventional methods. Baseline adjustments were more suited for the analysis of the upper extremity motor score compared to the spinal cord independence measure and its self-care subscore.

**Conclusions:**

The proposed baseline-adjusted proportional odds models allow the global treatment effect to be directly interpreted. This clear interpretation, the superior statistical power compared to the conventional analysis approaches, and the availability of open-source software support the application of this novel method for the analysis of ordinal outcomes of future clinical trials.

## Background

Statistical methods for the quantitative analysis of ordinal sum score outcomes in medical research are motivated by three distinct core ideas. There is currently no gold standard statistical method used for the analysis. Hence, the aim of this paper is to introduce an advanced tailored statistical method for the analysis of complex ordinal outcomes based on the advantages of the three existing core ideas. The novel method introduced in this paper is applicable to ordinal sum score measurements in general. However, it is here exemplified by an application based on two commonly used ordinal clinical outcome measurements from the field of spinal cord injury (SCI) research [1]. More precisely, the upper extremity motor score (UEMS) and the spinal cord independence measure (SCIM) serve as illustrative ordinal response variables to introduce and demonstrate the novel method. The UEMS and SCIM are frequently used ordinal clinical assessments to classify SCI patients.

SCIs result in life-long para- and tetraplegia, with high impact on the individual quality of life. To date, no treatment is available to regenerate the interrupted nerve fibers and repair the damaged spinal cord. Hence, patients with SCI basically only benefit from the rehabilitation programs that enable patients to compensate and adjust by maximizing their functional skills for the given neurological impairment caused by the SCI [2]. However, important progress has been made in the last 20 years in the scientific understanding of the processes regulating nerve fiber growth and regeneration.

Recent preclinical research in animal models has been extremely successful in demonstrating the efficacy of the antibody anti-Nogo-A raised against the axon growth inhibitory protein Nogo-A [3, 4]. These promising results warrant its application in patients suffering from acute spinal cord injury. The Nogo Inhibition in Spinal Cord Injury trial (NISCI, www.nisci-2020.eu, “To enhance plasticity, regeneration and functional recovery after acute spinal cord injury, a multicenter European clinical proof of concept trial”) will enroll tetraplegic patients with various degrees of complete to incomplete acute spinal cord injury in a double-blind, placebo-controlled trial to test the efficacy of this antibody therapy in improving motor outcome and quality of life. The newly developed statistical methodology, which quantifies treatment effects in trials with ordinal sum score outcomes, introduced in this paper also aims to improve the analysis of the NISCI trial.

### Current state of research

This section contrasts the three distinct statistical core ideas that are commonly used for the quantitative analysis of ordinal outcomes. As stated above, the novel method introduced in this paper is applicable to ordinal sum score measurements in general. However, the application is exemplified based on the commonly used ordinal clinical outcome measurements from the field of SCI research [1]:

- the total UEMS from 0 to 50 [5],
- the SCIM total score from 0 to 100 [6, 7], and
- the SCIM self-care (SCIMsc) subscore from 0 to 20 [6, 7].

In SCI research, it has been suggested that these sum score measurements correlate with changes in daily living activities that rely on recovery of upper extremity function [8–11]. UEMS measures the muscle contraction force for 10 key muscles on the arms and hands (5 on each body side), where each contributes a 6-point ordinal scale (0: total paralysis, through 5: active movement against full resistance). The score is not ordinal per se, because it can only be directly compared between patients with the same level of injury, that is, with the same pattern of uneffected key muscles (which, by definition, receive a 5 score). SCIM consists of sub-items assessing mobility abilities (bed, indoors and outdoor activities), respiration and sphincter management, and self-care (feeding, grooming, bathing, and dressing). Both, SCIM total score and self-care subscore, are considered ordinal and can be used to compare the independence of patients regardless of the corresponding level of injury.

The first core idea discussed is considered as the most prominent one. For this, the total sum score of the individual ordinal scores is treated as a conceptually continuous outcome measured on an interval scale. Classical statistical models or tests, such as baseline-adjusted analysis of covariance (ANCOVA) models or *t*-tests, are then applied to infer mean differences between treatment groups. The main advantages of this approach are ease of communication through reporting of difference in means and associated confidence intervals and the possibility of dealing with more complex trial designs, for example, through the incorporation of baseline information. However, Heller et al. [12] recommend to only apply linear regression and its variants for pragmatic analysis approaches for not highly skewed distributed outcome variables. In the case of the highly skewed UEMS and SCIM this does not seem adequate. Furthermore, differences in ordinal scores cannot directly be interpreted. For example, consider the case of two patients with very acute UEMSs, one with an UEMS of 25 and the other with an UEMS of 40. After six months, the UEMS of both patients increases by 5 points. Although the increase is the same for both patients, the relative percent recovery is much larger for the first patient from a medical point of view. As pointed out in [13], it is clearly erroneous to assume that important differences should be clinically equivalent across the whole scale [14].

This issue is dealt with by the remaining two core ideas, which respect the ordinal nature of the outcomes. One idea is to understand the contributions of each score item, i.e. SCIM item or each segment within the UEMS, as a multivariate ordinal outcome. Item-response analysis, most prominently with the Rasch model, tries to identify a latent continuous interval scale that generates the ordinal assessment. Item properties, such as unidimensionality and potential differential item functioning, have been assessed for the SCIM [15] and UEMS [16]. Once such a latent scale is identified, classical models and tests for continuous outcomes could be used to infer differences in means between treatment groups. The results, however, are difficult to interpret because treatment effects are reported on this latent scale. In SCI research, Reed et al. [17] recently introduced the spinal cord ability ruler (SCAR) as an interval construct to measure volitional performance after spinal cord injury. The SCAR is a single SCI measurement based on two existing and commonly used measurements (International Standards for Neurological Classification of SCI (ISNCSCI) and SCIM). The definition of the SCAR scores is based on a Rasch analysis and was validated by simulation with two databases. Additional statistical models which belong to the class of latent trait model for ordered polytomous responses are the Graded Response Model (GRM, [18]) as well as the Partial Credit Model (PCM, [19]).

The third idea is to define a simple treatment effect measure that can be directly interpreted on the original ordinal scale of the outcome measures. Tanadini et al. [8] suggest to compare the UEMS measurements with respect to the odds ratio (OR) of treatment versus control groups. The challenge here is to extend the classical proportional odds logistic regression Polr) model [20–22] for ordinal responses with a considerably high number of non-reducible outcome categories. Since the classical Polr model features one intercept parameter for each possible outcome category, this would require 50 intercept parameters for each possible stratum in a model for the UEMS sum score, which ranges between 0 and 50. Tanadini et al. [8] approach this problem by fitting one segment-wise proportional odds model. While this technique can be used for *p*-value-based inference of segment-wise ORs with potential baseline adjustment, it is impossible to derive a global treatment effect and a corresponding confidence interval. The latter information is, however, of utmost importance for communicating results of randomized clinical trials.

The above discussion stresses the need for an advanced tailored method for the analysis of complex ordinal outcomes that allows valid inference of a global interpretable treatment parameter and respects the original ordinal scale of the outcomes in potentially complex trial designs. We introduce a novel methodology that brings together the simplicity of a univariate sum score outcome and the interpretability of a global treatment effect. The method is based on maximum likelihood estimation and allows for baseline adjustment as well as confidence interval-based inference.

## Methods

The proposed baseline-adjusted proportional odds logistic regression model is generalizable and applicable for any statistical analysis with a conseriderably high amount of possible outcome categories. However, we here strengthen our argument by applying the method to specific ordinal sum score outcome variables from the field of SCI research. Thus, this section begins with the introduction to the underlying data set with which we later evaluate our models. We then focus on the specific models and their application in terms of statistical power and trial sample size calculations with the help of a simulation study. Furthermore, we present a concrete application of the method applied as an alternative analysis approach to the Sygen^®^ trial data.

### Data source and trial outcomes

The proposed treatment effect models were evaluated in a setting similar to the currently conducted NISCI trial. Patient data from the European Multicenter Study about Spinal Cord Injury (EMSCI, ClinicalTrials.gov Identifier: NCT01571531, www.emsci.org) were selected according to the NISCI inclusion criteria (age between 18 and 72 years; AIS of A, B, C or D; neurological level of injury between spinal segments C1 - C8 at time very acute; observed UEMS and SCIM at baseline, i.e. 2 - 28 days post-injury, and follow-up, i.e. 150 - 186 days post-injury). All patients gave written informed consent. In total, data on a subset of *N* = 350 female and male patients from EMSCI were obtained.

EMSCI tracks the functional and neurological recovery of patients during the first year after SCI. Patient data are collected in a highly standardized manner in 18 European centers during five specific time frames in reference to the day of injury:

- 0 for null (very acute, baseline). 0 - 15 days after the injury.
- I for acute I. 16 - 40 days after the injury.
- II for acute II. 70 - 98 days after the injury.
- III for acute III. 150 - 186 days after the injury.
- C for chronic. 300 - 546 days after the injury.

The drug efficacy being tested in a clinical trial is often quantified as the change of primary outcome between baseline and follow up. For the proceeding analysis, we therefore only consider the timepoints null with superscript 0 and time point acute III with superscript III. The models were evaluated with UEMS [5] and SCIM sum (sub)scores [6, 7] as outcomes. The UEMS total sum score at trial end point is abbreviated as M^III^. The symbols $\mathrm {S}_{\text {tot}}^{\text {III}}$ and $\mathrm {S}_{\text {sc}}^{\text {III}}$ denote the SCIM total sum and SCIM self-care subscore at time point acute III, respectively. Note that some of the subjects in the EMSCI database were assessed using SCIM-II [6], and others were assessed using SCIM-III [7]. However, for the purposes of our analysis, no distinction was made between the two versions of the measure because the self-care items of these measures are highly similar [7, 23].

### Enhanced proportional odds logistic regression (ePolr)

The enhanced proportional odds logistic regression (ePolr) model is an extension of the classical Polr model [20–22]. In the following, we elaborate the similarities of these two models as well as the enhanced properties of the ePolr model, i.e. the baseline-adjustment, stratification, and smoothing. For this, let y^III^ be a categorical response measured on an ordinal scale with a considerably high amount of *K* categories: $\mathrm {y}^{\text {III}} \in \{1, 2, \dots, K\}$. Moreover, y^0^ stands for the ordinal outcome variable measured at baseline, and *x* represents a univariate explanatory variable or, in the two-sample situation, *x*=0 for the control and *x*=1 for the active group. The models are formulated as models for conditional distribution functions:

$$\begin{array}{*{20}l} \mathbb{P} \big(\mathrm{Y}^{\text{III}} \leq \mathrm{y}^{\text{III}} \mid x) & = \text{expit} \big(h(\mathrm{y}^{\text{III}} \mid \mathrm{y}^{0}) + \beta \cdot x)  \\ \mathbb{P} \big(\mathrm{Y}^{\text{III}} \leq \mathrm{y}^{\text{III}} \mid \mathrm{y}^{0}, x) & = \text{expit} \big(h(\mathrm{y}^{\text{III}} \mid \mathrm{y}^{0}) + \beta \cdot x)  \end{array} $$

Both models use the inverse logit transformation / link function, also known as the expit transformation, to ensure interpretability of exp(*β*) as the conditional OR associated with a one unit increase in *x*↦*x*+1.

$$\begin{array}{*{20}l}{} \frac{\mathbb{P}(\mathrm{Y}^{\text{III}} \!\leq\! \mathrm{y}^{\text{III}} \!\mid\! \mathrm{y}^{0}, x+\!1)} {\mathbb{P}(\mathrm{Y}^{\text{III}} \!>\! \mathrm{y}^{\text{III}} \!\mid\! \mathrm{y}^{0}, x+\!1)}\!\left/\right.\!\frac{\mathbb{P}(\mathrm{Y}^{\text{III}} \!\leq\! \mathrm{y}^{\text{III}} \!\mid\! \mathrm{y}^{0}, x)} {\mathbb{P}(\mathrm{Y}^{\text{III}} \!>\! \mathrm{y}^{\text{III}} \!\mid\! \mathrm{y}^{0}, x)} =\! \exp(\beta) =\! \text{OR} \end{array} $$

The ePolr model is part of the transformation model family which was recently introduced by Hothorn et al. in [24]. Following this, the unknowns in these models that have to be estimated from the data are the regression coefficient *β* and the conditional transformation function *h*, which incorporates baseline information y^0^. In contrast to earlier proposals of similar models for the analysis of ordinal outcomes with a predefined transformation function *h* [25], we estimate this function from the data.

The classical Polr model [20–22] is based on a discrete parametrization of *h*, which assigns one intercept parameter $\theta _{k}, \; k \in \{1, 2, \dots, K-1\}$, to each possible outcome category without the possibility of stratifying with respect to baseline variables. For example in a SCI clinical trial, the number of required intercepts for the SCIM total sum score would be 100 and potentially exceeds the number of patients in such a trial. Although attempts to extend the classical model, and thus the discrete transformation function *h*, to many possible categories or even continuous outcomes have been reported [26], baseline adjustment is still a conceptual challenge. However, the incorporation of baseline information, such as y^0^, is very important in the context of clinical trials. To overcome this limitation, we introduce a smooth and parsimonious parametrization of the transformation *h* that allows for appropriate incorporation of baseline information as well as stratification by the variable strata:

$$\begin{array}{*{20}l}{} \mathbb{P} \big(\mathrm{Y}^{\text{III}} \!\leq\! \mathrm{y}^{\text{III}} \mid \mathrm{y}^{0}, \text{\texttt{strata}}, x) & = \text{expit} \big(h(\mathrm{y}^{\text{III}} \mid \mathrm{y}^{0}, \text{\texttt{strata}})\\ &\quad+ \beta \cdot x) \end{array} $$

The estimation of such a model with a considerable high number of outcome categories *K* and in the presence of strata, the number of intercept parameters is equal to *K*−1 times the number of strata. In such a situation, however, one does not expect abrupt jumps between intercepts of adjacent categories. Therefore, the replacement of category-specific intercepts with a smooth function *h* depending on a few parameters only was suggested by Parsons [13]. The latter contribution developed corresponding proportional odds models for repeated measurements using a generalised estimating equations (GEE) approach. Parsons’ method features orthogonal polynomials of different degrees for *h* and requires to expand the data by the number of categories. Stratification, i.e. allowing different transformation functions *h* for different strata, would be conceptually possible if a further expansion of the data by the number of strata is feasible. Similar approaches for the estimation of conditional distribution functions by means of data expansion have been suggested in other contexts as well [27]. Here, we follow Hothorn et al. [24] and employ a model parameterization allowing model estimation and inference in the maximum likelihood framework, also in the presence of large *K* and potentially many strata. For this, we introduce a spline polynomial $a(y)^{\top } \vartheta : \mathbb {R} \rightarrow \mathbb {R}$ in terms of *P*≪*K* basis functions and parameterize the transformation function as *h*(*y*)=*a*(⌊*y*⌋)^⊤^*𝜗*, with ⌊*y*⌋ being the largest integer smaller or equal some real value *y*. A numerically attractive choice for such a spline is a Bernstein polynomial on the interval [1,*K*] [24]. Monotonicity of *h* can be ensured by a linear constraint on the *P* parameters $\vartheta = (\vartheta _{1} < \dots < \vartheta _{P})$. Stratas are incorporated by application of this basis to each stratum $a(y) \otimes (0, \dots, 1, \dots, 0)$, the second factor being a dummy coding of strata.

In the specific case of a stratified ePolr model, the likelihood contribution of a single observation with variable y^0^ at baseline, y^III^ at trial end and strata strata is

$$\begin{array}{*{20}l}{} \text{expit} \big(h(\mathrm{y}^{\text{III}} \mid \mathrm{y}^{0}, &\; \text{\texttt{strata}}) + \beta \cdot x \big) \\ & - \text{expit} \big(h(\mathrm{y}^{\text{III}} - 1 \mid \mathrm{y}^{0},\! \; \text{\texttt{strata}}) + \beta\! \cdot x \big) \end{array} $$

under the conventions *h*(0)=−*∞* and *h*(*K*)=*∞*. Here, the transformation function *h* is stratified by the y^0^ measurements taken at baseline and the stratification variable strata. For each stratum, we obtain a stratum-specific baseline log-odds function and a response-varying effect of y^0^, the outcome at baseline. Note that all resulting strata have a direct impact on the conditional distribution and all moments, such as means, variances, skewness, and kurtosis, might vary depending on strata. Hence, the baseline-adjusted ePolr model allows for parametric prediction methods. The model parameters *β* and *h* are simultaneously estimated by maximum likelihood [24] with the help of the R package tram [28].

### ePolr models for spinal cord injury clinical trials

For the evaluation of the suggested ePolr model, we specifically tailored three models M1–3 to SCI related clinical trial outcomes:

- M1: UEMS sum score M^III^ from 0 to 50,
- M2: SCIM sum score $\mathrm {S}_{\text {tot}}^{\text {III}}$ from 0 to 100,
- M3: SCIM self-care subscore $\mathrm {S}_{\text {sc}}^{\text {III}}$ from 0 to 20.

For the sake of simplicity, we considered a two-arm trial that compares a control (*I*(trt) = 0) to a novel treatment (*I*(trt) = 1). Each of the following models describes the conditional cumulative distribution function of the corresponding sum score as a function of baseline variables (mainly the baseline sum scores at time 0 as well as the number of segments below level of injury for the UEMS) and a global treatment effect *β*_trt_:

$$\begin{array}{*{20}l}{}\mathbb{P} \big(\mathrm{M}^{\text{III}} \!\leq\! \mathrm{m}^{\text{III}} \!\mid\! \mathrm{m}^{0}, \text{\texttt{\#seg}}, \text{\texttt{trt}} \big) & \,=\, \text{expit} \big(h(\mathrm{m}^{\text{III}} \mid \mathrm{m}^{0}, {\text{\texttt{\#seg}}})\\ &\quad+ \beta_{\text{\texttt{trt}}} \cdot I(\text{\texttt{trt}}) \big)  \end{array} $$

$$\begin{array}{*{20}l}{} \mathbb{P} \big(\mathrm{S}_{\text{tot}}^{\text{III}} \!\leq\! \mathrm{s}_{\text{tot}}^{\text{III}} \!\mid\! \mathrm{s}_{\text{tot}}^{0}, \text{\texttt{trt}} \big) & \,=\, \text{expit} \big(h(\mathrm{s}_{\text{tot}}^{\text{III}} \!\mid\! \mathrm{s}_{\text{tot}}^{0}) + \beta_{\text{\texttt{trt}}} \!\cdot\! I(\text{\texttt{trt}}) \big)\\ &  \end{array} $$

$$\begin{array}{*{20}l}{} \mathbb{P} \big(\mathrm{S}_{\text{sc}}^{\text{III}} \leq \mathrm{s}_{\text{sc}}^{\text{III}} \mid \mathrm{s}_{\text{sc}}^{0}, \text{\texttt{trt}} \big) & \,=\, \text{expit} \big(h(\mathrm{s}_{\text{sc}}^{\text{III}} \!\mid\! \mathrm{s}_{\text{sc}}^{0}) + \beta_{\text{\texttt{trt}}} \!\cdot I(\text{\texttt{trt}}) \big)    \end{array} $$

As mentioned previously, the inverse logit transformation, also known as the expit transformation, ensures interpretability of the treatment effect exp(*β*_trt_) as the conditional odds ratio that compares the treatment group and the control group given baseline variables. For the UEMS, this odds ratio is given by

$$\begin{array}{*{20}l}{} \frac{\mathbb{P}(\mathrm{M}^{\text{III}} \!\leq\! \mathrm{m}^{\text{III}} \mid \mathrm{m}^{0}, \text{\texttt{\#seg}}, \text{active})} {\mathbb{P}(\mathrm{M}^{\text{III}} \!>\! \mathrm{m}^{\text{III}} \mid \mathrm{m}^{0}, \text{\texttt{\#seg}},\text{active})}\left/\right. {\frac{\mathbb{P}(\mathrm{M}^{\text{III}} \leq \mathrm{m}^{\text{III}} \mid \mathrm{m}^{0}, \text{\texttt{\#seg}},\text{control})} {\mathbb{P}(\mathrm{M}^{\text{III}} > \mathrm{m}^{\text{III}} \mid \mathrm{m}^{0}, \text{\texttt{\#seg}}, \text{control})}}\\ = \exp(\beta_{\text{trt}}).{\kern75pt} \end{array} $$

The variable #seg is defined as the number of left and right spinal segments below motor level that are at or more caudal than C5 and at or more rostral than T1. Stratification with respect to #seg is of utmost importance for UEMS, because the UEMS total score can only be considered as an ordinal variable when comparing patients with the same level of injury [8]. The parameter exp(*β*_trt_) for the two SCIM models M2 and M3 can be interpreted in the same way. The unknowns in these models that have to be estimated from the patient trial data are the treatment effect *β*_trt_ and the conditional transformation function *h*, which incorporates baseline information. In the specific case of model M1, the likelihood contribution of a treated patient with a UEMS total sum score m^0^ at baseline, m^III^ after six months and #seg segments below motor level is

$$\begin{array}{*{20}l} \text{expit} \big(h(\mathrm{m}^{\text{III}} \mid \mathrm{m}^{0}, & \text{\texttt{\#seg}}) + \beta_{\text{\texttt{trt}}} \cdot I(\text{\texttt{trt}}) \big) \\ & - \text{expit} \big(h(\mathrm{m}^{\text{III}} - 1 \mid \mathrm{m}^{0}, \text{\texttt{\#seg}})\\ &+ \beta_{\text{\texttt{trt}}} \cdot I(\text{\texttt{trt}}) \big). \end{array} $$

under the conventions *h*(0)=−*∞* and *h*(*K*)=*∞*.

The specific choice of the variables considered for model M1 reflects that recovery in the upper extremities of patients with cervical SCI is not only dependent on the severity of the injury (as measured by the baseline UEMS) but also on the injury level [29, 30]. Hence, the transformation function *h* in model M1 is stratified by the UEMS total sum score measurements observed at baseline, m^0^, and the number of tested segments below motor level, #seg, representing the injury level. The extracted data set from the EMSCI database has observations with four to 10 segments below motor level #seg that are at or more caudal than C5 and at or more rostral than T1. In addition, the UEMS at baseline ranges between 0 and 28. Consequently, we defined three strata ([0,6],[7,8],[9,10]) for #seg and estimated a stratum-specific response-varying effect in the UEMS baseline values. More specifically, the spline basis reads *a*(*y*)×(1,0,0)×(1,m^0^) for stratum #seg∈[0,6],*a*(*y*)×(0,1,0)×(1,m^0^) for stratum #seg∈[7,8], and *a*(*y*)×(0,0,1)×(1,m^0^) for the last stratum. For each stratum, we obtain a stratum-specific baseline log-odds function and a response-varying effect of m^0^, the outcome at baseline. For our empirical experiments, we chose seven parameters for the Bernstein polynomial parametrization [24] on the interval [0,50] and thus estimated a total of 7×3×2=42 parameters for the conditional transformation function *h*. Due to the monotonicity constraint, the effective degrees of freedom for *h* is, however, much smaller than the corresponding number of parameters.

The estimated baseline-adjusted ePolr models allow for parametric prediction methods such that a direct comparison between treated and untreated patients is straightforward. The model parameters are simultaneously estimated by maximum likelihood [24]. Concurrently, the OR exp(*β*_trt_), along with a variability assessment and thus a confidence interval, is directly estimated by the model. Corresponding *p*-values for testing the two-sided null hypothesis of no treatment effect (*β*_trt_ = 0) rely on the asymptotic normality of the maximum likelihood estimator. Hereafter, we will refer to the Wald test as the asymptotic ePolr test, short for asymptotic enhanced baseline-adjusted proportional odds regression coefficient test.

For small samples, however, the asymptotic ePolr test for the treatment effect *β*_trt_ might be too liberal because of lack of approximation accuracy. We therefore in addition applied a model-based permutation test [31, 32] for *β*_trt_ = 0. A general theory for such permutation tests is available in [33, 34] and can be directly applied to the context studied here [35]. The null hypothesis of no treatment effect (*β*_trt_ = 0) implies that the distribution of the model scores for the treatment effect *β*_trt_ in models M1–3 is the same in the control and treatment group. For this, we first estimated the intercept-only models based on model M1, M2, and M3 and extracted the single score contributions *S*_*i*_ of each observation *i* for each of the models. More specifically, the score *S*_*i*_ is defined as the derivative

$$\begin{array}{@{}rcl@{}} S_{i} = \left. \frac{\partial \ell_{i}(\alpha)}{\partial \alpha}\right|_{\alpha = 0} \end{array} $$

of the log-likelihood contribution of the *i*th subject under the null of an absent treatment effect

$$\begin{array}{*{20}l} \ell_{i}(\alpha) = & \log\left(\mathbb{P} \left(\mathrm{M}^{\text{III}} = \mathrm{m}^{\text{III}}_{i} \mid \mathrm{m}^{0}_{i}, \text{\texttt{\#seg}}_{i} \right)\right) \\ = & \log\left(\text{expit} \left(h\left(\mathrm{m}^{\text{III}}_{i} \mid \mathrm{m}^{0}_{i}, \text{\texttt{\#seg}}_{i}\right) + \alpha \right)\right.\\ &\left. - \text{expit} \left(h\left(\mathrm{m}^{\text{III}}_{i} - 1 \mid \mathrm{m}^{0}_{i}, \text{\texttt{\#seg}}_{i}\right) + \alpha \right)\right). \end{array} $$

The resulting test statistics $T_{1, 2, 3}~=~\sum _{i=1}^{N} S_{i} \cdot I(\text {\texttt {trt}})$ is the sum of the score contributions *S*_*i*_ of the observations allocated to the treatment group. There is a strong connection to the Wilcoxon rank sum test: Without stratification and application of the transformation function *h*(m*i*III)=logit(*N*^−1^*R*_*i*_) with upranks $R_{i}, \; i = 1, \dots, N$, the scores *S*_*i*_ are the Wilcoxon scores [36]. The fact that both the permutation test suggested here as well as the Wilcoxon rank sum test can be derived as score tests in a proportional odds model highlights their similarity and explains why one can expect both procedures to be especially powerful against proportional odds alternatives.

The conditional null distribution of the test statistic is approximated by 10,000 permutations that rearrange the labels for arm allocation and then are used to obtain the permutation *p*-value. Hereafter, we will refer to this model-based permutation test of the score contributions as permutated ePolr test.

### Sample size and statistical power calculation

Based on the previously defined models M1–3, we introduce a procedure that allows for a simulation-based assessment of an appropriate number of patients to be enrolled in a trial. In addition, we used the simulations to compare the power of our model-based inference method to the power of conventional test procedures for total sum scores.

Our simulation of the placebo-controlled randomized clinical trials with two arms based on the EMSCI data set has the following specific levels of experimental conditions:

- total sample size (N): 80, 120, 160, 200, 240
- treatment effect exp(*β*_trt_) ([OR]): 1, 1.25, 1.5, 1.75, 2, 2.25, 2.5, 2.75, 3

The combinations of these five trial sample sizes and nine possible treatment effects result in 45 different trial scenarios. For each scenario, we sampled *N* EMSCI trial participants and randomly allocated them to control or treatment groups following a 1:1 allocation scheme.

By restricting the treatment effect to *β*_trt_ = 0 in the models M1–3, one describes the spontaneous neurological recovery for patients under standard of care, i.e. patients in the control group. In the first step, the models, and thus the stratified transformation functions *h*, were estimated from treatment-naive EMSCI patient data with spontaneous neurological recovery patterns under standard of care. *β*_trt_ is constantly set to 0 for participants in the control group. In the second step, the outcomes $\mathrm {m}^{\text {III}}, \mathrm {s}_{\text {tot}}^{\text {III}}, \mathrm {s}_{\text {sc}}^{\text {III}}$ for the treated patients were simulated by a standard parametric bootstrap procedure sampling new outcomes under the specific postulated treatment effects exp(*β*_trt_) ([OR]): 1,1.25,1.5,1.75,2,…,3. The baseline measurements at time point 0 ($\mathrm {m}^{0}, \mathrm {s}_{\text {tot}}^{0}, \mathrm {s}_{\text {sc}}^{0}$) were left unchanged and hence are equivalent to the original observations from the EMSCI data.

Concurrently, a battery of five different significance tests for testing *H*_0_:*β*_trt_ = 0 were applied to each simulated trial, and subsequently the statistical power was evaluated. The statistical power $\mathbb {P}$(reject *H*_0_∣*β*_trt_>0) was estimated as the fraction of significant *p*-values for treatment effect *β*_trt_ at the nominal level *α* = 0.05 among the 15,000 replications of the 45 different trial scenarios.

As noted above, we evaluated the new method by applying it to three different outcomes: UEMS total sum scores m^III^, SCIM total sum scores $\mathrm {S}_{\text {tot}}^{\text {III}}$, and SCIM self-care subscore $\mathrm {S}_{\text {sc}}^{\text {III}}$. Subsequently, the treatment effects for these outcomes were tested with the following statistical tests:

- *t*-test comparing the difference of the total sum score at time III and the total sum score at time 0 between the two treatment groups
- Wilcoxon rank sum test
- ANCOVA comparing the difference between the two treatment groups at time III while adjusting each patient’s follow-up score for his or her baseline score at time 0 [37]
- Asymptotic ePolr test based on models M1, M2, and M3
- Permutated ePolr test based on models M1, M2, and M3

### Alternative analysis of the sygen^®^ trial

As a concrete application of model M1, we reanalyzed a data subset of the Sygen^®^ trial; the study design is explained in detail elsewhere [38–40]. Briefly, in this trial, *N* = 760 SCI participants from 28 centers in North America were recruited over a 5-year time period between 1992 and 1997. The primary outcome was a dichotomized variable derived from an ordinal scale representing the overall neurological status of a patient (see [39] for the exact definition). Subsequently, the primary outcome was analyzed by means of binary logistic regression.

Another reanalysis of a data subsample of the study was recently published [8]. For this, the authors redefined the single-score UEMS as the primary outcome of the trial and applied the autoregressive transitional ordinal model to test for treatment effect. Following this, we also redefined the primary trial outcome. Based on model M1, we used the UEMS total sum score m^III^ observed at time acute III as the primary trial outcome. The alternative analysis of the trial subsample data set specifically concentrates on the UEMS total sum score observations because the original data collection did not incorporate the SCIM measurements.

Following our simulation study, we extracted the patient data from the Sygen^®^ trial according to the previously mentioned inclusion criteria of the NISCI trial. The original Sygen^®^ trial had two treatment doses at the beginning; the higher dosage was abandoned during the study. Consequently, only patients treated with the lower dosage were considered here. The resulting subsample of *N* = 335 patients are from 26 different study centers.

This analysis is intended to give a practical example of an application of the enhanced baseline-adjusted proportional odds model and should not be considered as a definitive conclusion about the quantification of treatment effect or the outcome of the Sygen^®^ trial in general.

## Results

### Randomized clinical trial simulation

The purpose of the simulation study was to compare our novel method with conventional analysis methods, such as the *t*-test, Wilcoxon rank sum test, and ANCOVA. Note that the simulation setting as well as the results reported are based on ordinal outcome measurements specifically tailored for clinical assessment and classification of SCI patients. However, the model setup is generalizable to any type of ordinal response measure with a considerably high number of outcome categories.

Tables 1, 2, and 3 report the estimated statistical power of models M1, M2, and M3, respectively. The newly introduced permutated ePolr test outperformed conventional analysis methods in every simulation setup. Especially in trial setups with relatively small sample sizes, the results of the significance test based on the baseline-adjusted proportional odds regression model had clear advantages with regards to power, e.g. in detecting significant treatment effects. In all three tables, the estimated statistical power of the asymptotic ePolr test and the permutated ePolr test is consistently higher than the estimates from conventional approaches, i.e. the *t*-test, Wilcoxon rank sum test, and ANCOVA. However, for extreme experimental conditions, e.g. large postulated treatment effects such as exp(*β*_trt_) = 2.75, the differences among the approaches diminished.

**Table 1:** Statistical power of the UEMS total sum score model M1 for all simulation settings (1:1 allocation) compared with that of conventional approaches

**Experimental conditions** (1:1 allocation)				**Novel model-based methods**		**Conventional approaches**		
N total	N trtmt	N ctrl	Odds ratio	Asymptotic ePolr test based on model M1	Permutated ePolr test based on model M1	*t*-test M1	Wilcoxon rank sum test M1	ANCOVA M1
80	40	40	1.00	.062 [.058,.067]	.049 [.045,.052]	.049 [.046,.053]	.049 [.045,.052]	.048 [.045,.052]
120	60	60	1.00	.061 [.057,.065]	.050 [.046,.053]	.053 [.049,.056]	.050 [.046,.053]	.052 [.049,.056]
160	80	80	1.00	.058 [.054,.062]	.046 [.042,.049]	.049 [.046,.053]	.048 [.045,.051]	.049 [.045,.052]
200	100	100	1.00	.057 [.054,.061]	.048 [.045,.052]	.048 [.044,.051]	.047 [.044,.051]	.048 [.045,.052]
240	120	120	1.00	.061 [.058,.065]	.053 [.049,.056]	.053 [.049,.056]	.052 [.048,.055]	.052 [.049,.056]
80	40	40	1.25	.105 [.099,.111]	.086 [.082,.091]	.077 [.073,.082]	.074 [.070,.078]	.079 [.074,.083]
120	60	60	1.25	.124 [.119,.130]	.103 [.098,.108]	.091 [.086,.095]	.089 [.084,.093]	.091 [.086,.095]
160	80	80	1.25	.142 [.137,.148]	.122 [.117,.128]	.103 [.098,.108]	.104 [.099,.109]	.106 [.101,.111]
200	100	100	1.25	.162 [.156,.168]	.142 [.136,.148]	.124 [.119,.129]	.122 [.117,.128]	.127 [.122,.132]
240	120	120	1.25	.182 [.176,.189]	.162 [.156,.168]	.136 [.131,.142]	.135 [.130,.141]	.139 [.133,.145]
80	40	40	1.50	.194 [.186,.202]	.164 [.158,.170]	.145 [.139,.151]	.143 [.137,.148]	.146 [.140,.151]
120	60	60	1.50	.265 [.258,.273]	.233 [.227,.240]	.194 [.188,.200]	.195 [.189,.202]	.199 [.193,.205]
160	80	80	1.50	.328 [.321,.336]	.298 [.290,.305]	.243 [.236,.250]	.241 [.234,.248]	.246 [.239,.253]
200	100	100	1.50	.393 [.385,.401]	.364 [.356,.371]	.289 [.281,.296]	.290 [.282,.297]	.296 [.288,.303]
240	120	120	1.50	.447 [.439,.455]	.416 [.408,.424]	.331 [.324,.339]	.334 [.327,.342]	.339 [.332,.347]
80	40	40	1.75	.294 [.285,.303]	.260 [.253,.268]	.225 [.218,.232]	.221 [.214,.228]	.227 [.221,.234]
120	60	60	1.75	.424 [.415,.432]	.384 [.376,.392]	.325 [.318,.333]	.321 [.314,.329]	.327 [.320,.335]
160	80	80	1.75	.533 [.525,.541]	.497 [.489,.505]	.408 [.400,.416]	.407 [.399,.415]	.414 [.406,.422]
200	100	100	1.75	.630 [.622,.638]	.596 [.589,.604]	.492 [.484,.500]	.494 [.486,.502]	.499 [.491,.507]
240	120	120	1.75	.709 [.701,.716]	.678 [.670,.685]	.569 [.561,.577]	.575 [.567,.583]	.576 [.568,.584]
80	40	40	2.00	.430 [.420,.440]	.383 [.375,.391]	.320 [.312,.327]	.315 [.308,.323]	.323 [.315,.330]
120	60	60	2.00	.586 [.578,.595]	.543 [.535,.551]	.458 [.450,.466]	.460 [.452,.468]	.462 [.454,.470]
160	80	80	2.00	.719 [.712,.727]	.685 [.677,.692]	.574 [.566,.582]	.579 [.571,.587]	.580 [.572,.588]
200	100	100	2.00	.805 [.799,.812]	.782 [.776,.789]	.676 [.668,.683]	.681 [.673,.688]	.681 [.673,.688]
240	120	120	2.00	.862 [.856,.867]	.846 [.840,.852]	.750 [.743,.757]	.753 [.746,.760]	.756 [.749,.763]
80	40	40	2.25	.534 [.524,.543]	.488 [.480,.496]	.428 [.420,.436]	.421 [.413,.429]	.426 [.418,.434]
120	60	60	2.25	.717 [.709,.724]	.678 [.671,.686]	.588 [.581,.596]	.591 [.583,.599]	.591 [.583,.599]
160	80	80	2.25	.832 [.826,.838]	.809 [.803,.815]	.707 [.700,.715]	.710 [.703,.717]	.712 [.704,.719]
200	100	100	2.25	.905 [.900,.909]	.887 [.882,.892]	.797 [.791,.804]	.801 [.795,.808]	.802 [.795,.808]
240	120	120	2.25	.950 [.946,.953]	.941 [.937,.945]	.873 [.868,.879]	.876 [.871,.881]	.877 [.871,.882]
80	40	40	2.50	.637 [.627,.646]	.588 [.580,.595]	.509 [.501,.517]	.504 [.496,.512]	.508 [.500,.516]
120	60	60	2.50	.816 [.809,.822]	.783 [.776,.789]	.689 [.682,.697]	.694 [.686,.701]	.693 [.685,.700]
160	80	80	2.50	.909 [.904,.913]	.892 [.887,.897]	.807 [.801,.814]	.808 [.802,.815]	.813 [.807,.819]
200	100	100	2.50	.957 [.954,.960]	.948 [.944,.951]	.888 [.882,.893]	.889 [.883,.894]	.891 [.885,.895]
240	120	120	2.50	.983 [.981,.985]	.979 [.977,.982]	.934 [.930,.938]	.936 [.932,.940]	.936 [.932,.940]
80	40	40	2.75	.715 [.706,.724]	.677 [.669,.684]	.597 [.590,.605]	.591 [.583,.599]	.599 [.591,.607]
120	60	60	2.75	.881 [.875,.886]	.856 [.850,.862]	.775 [.768,.782]	.776 [.769,.783]	.777 [.771,.784]
160	80	80	2.75	.952 [.948,.955]	.941 [.937,.945]	.884 [.879,.889]	.886 [.881,.891]	.888 [.882,.893]
200	100	100	2.75	.981 [.979,.983]	.977 [.975,.979]	.937 [.933,.941]	.940 [.936,.944]	.940 [.936,.944]
240	120	120	2.75	.994 [.992,.995]	.992 [.990,.993]	.972 [.969,.975]	.973 [.970,.975]	.974 [.972,.977]
80	40	40	3.00	.781 [.773,.789]	.743 [.736,.750]	.665 [.657,.672]	.660 [.653,.668]	.665 [.657,.673]
120	60	60	3.00	.926 [.922,.930]	.908 [.903,.913]	.841 [.835,.847]	.841 [.835,.847]	.843 [.837,.849]
160	80	80	3.00	.975 [.973,.978]	.968 [.965,.971]	.929 [.925,.933]	.929 [.925,.933]	.930 [.926,.934]
200	100	100	3.00	.992 [.991,.994]	.990 [.988,.992]	.967 [.964,.969]	.968 [.965,.970]	.969 [.966,.972]
240	120	120	3.00	.998 [.997,.999]	.997 [.997,.998]	.987 [.985,.989]	.988 [.986,.989]	.988 [.986,.989]

Point estimates of the statistical power and 95% Wilson confidence intervals are reported

**Table 2:** Statistical power of the SCIM total sum score model M2 for all simulation settings (1:1 allocation) compared with that of conventional approaches

**Experimental conditions** (1:1 allocation)				**Novel model-based methods**		**Conventional approaches**		
N total	N trtmt	N ctrl	Odds ratio	Asymptotic ePolr test based on model M2	Permutated ePolr test based on model M2	*t*-test M2	Wilcoxon rank sum test M2	ANCOVA M2
80	40	40	1.00	.060 [.056,.063]	.048 [.044,.051]	.049 [.046,.053]	.047 [.044,.051]	.049 [.045,.052]
120	60	60	1.00	.064 [.060,.068]	.053 [.050,.057]	.055 [.051,.059]	.053 [.050,.057]	.052 [.049,.056]
160	80	80	1.00	.059 [.055,.063]	.051 [.047,.054]	.051 [.048,.055]	.050 [.046,.053]	.050 [.047,.054]
200	100	100	1.00	.058 [.055,.062]	.050 [.047,.054]	.049 [.045,.052]	.051 [.047,.054]	.050 [.046,.053]
240	120	120	1.00	.055 [.052,.059]	.048 [.045,.052]	.050 [.046,.053]	.050 [.047,.054]	.049 [.045,.052]
80	40	40	1.25	.099 [.094,.104]	.084 [.079,.088]	.078 [.074,.082]	.076 [.072,.080]	.080 [.075,.084]
120	60	60	1.25	.123 [.118,.129]	.108 [.103,.113]	.095 [.091,.100]	.099 [.094,.104]	.094 [.089,.099]
160	80	80	1.25	.140 [.134,.145]	.125 [.120,.130]	.105 [.101,.110]	.110 [.105,.115]	.106 [.101,.111]
200	100	100	1.25	.161 [.155,.167]	.144 [.138,.150]	.120 [.115,.126]	.126 [.121,.131]	.123 [.118,.129]
240	120	120	1.25	.187 [.181,.193]	.172 [.166,.178]	.139 [.133,.144]	.149 [.143,.154]	.141 [.135,.147]
80	40	40	1.50	.196 [.189,.202]	.170 [.164,.177]	.148 [.142,.154]	.151 [.146,.157]	.149 [.143,.155]
120	60	60	1.50	.265 [.258,.273]	.238 [.232,.245]	.196 [.190,.202]	.205 [.198,.211]	.201 [.195,.208]
160	80	80	1.50	.338 [.330,.346]	.315 [.307,.322]	.253 [.246,.260]	.268 [.261,.275]	.261 [.254,.268]
200	100	100	1.50	.403 [.395,.410]	.377 [.369,.384]	.308 [.301,.315]	.323 [.315,.330]	.310 [.303,.318]
240	120	120	1.50	.465 [.457,.473]	.439 [.431,.447]	.355 [.347,.363]	.377 [.369,.385]	.368 [.360,.375]
80	40	40	1.75	.317 [.310,.325]	.286 [.278,.293]	.241 [.234,.248]	.248 [.241,.255]	.247 [.240,.254]
120	60	60	1.75	.444 [.436,.452]	.410 [.402,.418]	.342 [.334,.350]	.355 [.348,.363]	.345 [.337,.352]
160	80	80	1.75	.542 [.534,.550]	.510 [.502,.518]	.428 [.420,.436]	.448 [.440,.456]	.437 [.429,.445]
200	100	100	1.75	.640 [.633,.648]	.617 [.609,.624]	.517 [.509,.525]	.541 [.533,.549]	.527 [.519,.535]
240	120	120	1.75	.718 [.711,.726]	.696 [.689,.704]	.590 [.582,.597]	.620 [.612,.628]	.605 [.597,.613]
80	40	40	2.00	.446 [.438,.454]	.404 [.396,.412]	.344 [.336,.351]	.355 [.348,.363]	.356 [.348,.364]
120	60	60	2.00	.605 [.597,.613]	.572 [.564,.580]	.487 [.479,.495]	.507 [.499,.515]	.495 [.487,.503]
160	80	80	2.00	.722 [.715,.729]	.697 [.689,.704]	.598 [.590,.605]	.618 [.610,.626]	.610 [.602,.617]
200	100	100	2.00	.817 [.811,.824]	.799 [.793,.805]	.703 [.695,.710]	.729 [.722,.736]	.714 [.707,.722]
240	120	120	2.00	.873 [.867,.878]	.859 [.853,.864]	.772 [.765,.778]	.796 [.789,.802]	.780 [.773,.786]
80	40	40	2.25	.566 [.558,.574]	.526 [.518,.534]	.449 [.441,.457]	.462 [.454,.470]	.461 [.453,.469]
120	60	60	2.25	.728 [.721,.736]	.700 [.693,.708]	.606 [.598,.614]	.624 [.616,.632]	.619 [.611,.627]
160	80	80	2.25	.847 [.841,.853]	.827 [.821,.833]	.736 [.729,.743]	.755 [.748,.762]	.749 [.742,.756]
200	100	100	2.25	.916 [.911,.920]	.904 [.899,.909]	.829 [.823,.835]	.849 [.843,.854]	.841 [.835,.846]
240	120	120	2.25	.951 [.947,.954]	.943 [.940,.947]	.885 [.879,.890]	.899 [.894,.904]	.893 [.888,.898]
80	40	40	2.50	.660 [.653,.668]	.624 [.616,.631]	.538 [.530,.546]	.554 [.546,.562]	.554 [.546,.562]
120	60	60	2.50	.828 [.822,.834]	.806 [.800,.813]	.713 [.706,.721]	.735 [.728,.742]	.728 [.720,.735]
160	80	80	2.50	.915 [.911,.920]	.903 [.898,.908]	.834 [.828,.840]	.852 [.846,.857]	.843 [.837,.849]
200	100	100	2.50	.959 [.956,.962]	.953 [.949,.956]	.903 [.898,.908]	.918 [.913,.922]	.912 [.907,.917]
240	120	120	2.50	.983 [.981,.985]	.980 [.978,.982]	.950 [.946,.953]	.958 [.954,.961]	.955 [.952,.959]
80	40	40	2.75	.747 [.739,.753]	.710 [.703,.717]	.626 [.618,.634]	.636 [.628,.643]	.641 [.633,.648]
120	60	60	2.75	.891 [.886,.896]	.872 [.866,.877]	.799 [.793,.806]	.817 [.810,.823]	.809 [.803,.816]
160	80	80	2.75	.958 [.954,.961]	.949 [.945,.952]	.896 [.891,.901]	.905 [.901,.910]	.904 [.899,.909]
200	100	100	2.75	.985 [.983,.987]	.982 [.979,.984]	.950 [.947,.954]	.959 [.955,.962]	.955 [.951,.958]
240	120	120	2.75	.995 [.994,.996]	.994 [.992,.995]	.977 [.974,.979]	.981 [.979,.984]	.980 [.978,.982]
80	40	40	3.00	.805 [.798,.811]	.776 [.769,.783]	.700 [.693,.708]	.707 [.700,.715]	.710 [.702,.717]
120	60	60	3.00	.934 [.930,.938]	.923 [.919,.928]	.867 [.861,.872]	.879 [.874,.885]	.873 [.868,.879]
160	80	80	3.00	.979 [.977,.982]	.975 [.973,.978]	.945 [.941,.949]	.951 [.947,.954]	.949 [.946,.953]
200	100	100	3.00	.993 [.991,.994]	.992 [.990,.993]	.978 [.976,.981]	.981 [.979,.983]	.981 [.978,.983]
240	120	120	3.00	.998 [.997,.999]	.998 [.997,.999]	.991 [.989,.992]	.994 [.992,.995]	.993 [.991,.994]

Point estimates of the statistical power and 95% Wilson confidence intervals are reported

**Table 3:** Statistical power of the SCIM self-care subscore model M3 for all simulation settings (1:1 allocation) compared with that of conventional approaches

**Experimental conditions** (1:1 allocation)				**Novel model-based methods**		**Conventional approaches**		
N total	N trtmt	N ctrl	Odds ratio	Asymptotic ePolr test based on model M3	Permutated ePolr test based on model M3	*t*-test M3	Wilcoxon rank sum test M3	ANCOVA M3
80	40	40	1.00	.071 [.067,.075]	.050 [.046,.053]	.050 [.046,.053]	.049 [.046,.053]	.050 [.046,.053]
120	60	60	1.00	.071 [.067,.075]	.052 [.049,.056]	.052 [.049,.056]	.054 [.051,.058]	.052 [.049,.056]
160	80	80	1.00	.068 [.064,.072]	.050 [.046,.053]	.051 [.047,.055]	.049 [.046,.053]	.050 [.047,.054]
200	100	100	1.00	.069 [.065,.073]	.051 [.048,.055]	.051 [.047,.055]	.051 [.048,.055]	.050 [.047,.054]
240	120	120	1.00	.067 [.064,.072]	.049 [.045,.052]	.050 [.047,.054]	.049 [.046,.053]	.050 [.047,.054]
80	40	40	1.25	.113 [.108,.119]	.087 [.083,.092]	.080 [.076,.084]	.079 [.075,.084]	.080 [.076,.085]
120	60	60	1.25	.136 [.131,.142]	.108 [.103,.113]	.096 [.092,.101]	.099 [.094,.104]	.098 [.093,.102]
160	80	80	1.25	.152 [.147,.158]	.122 [.116,.127]	.109 [.104,.114]	.113 [.108,.118]	.108 [.103,.113]
200	100	100	1.25	.178 [.172,.184]	.142 [.136,.147]	.124 [.118,.129]	.128 [.123,.134]	.125 [.120,.131]
240	120	120	1.25	.205 [.199,.212]	.168 [.162,.174]	.149 [.144,.155]	.151 [.145,.156]	.150 [.144,.155]
80	40	40	1.50	.212 [.206,.219]	.172 [.166,.178]	.151 [.146,.157]	.155 [.149,.161]	.152 [.146,.158]
120	60	60	1.50	.285 [.278,.292]	.237 [.230,.244]	.205 [.199,.212]	.211 [.205,.218]	.207 [.200,.213]
160	80	80	1.50	.357 [.349,.364]	.306 [.299,.314]	.263 [.256,.270]	.270 [.263,.277]	.263 [.256,.270]
200	100	100	1.50	.422 [.414,.430]	.369 [.361,.376]	.313 [.306,.321]	.325 [.317,.332]	.314 [.306,.321]
240	120	120	1.50	.482 [.474,.490]	.429 [.421,.436]	.366 [.358,.373]	.375 [.367,.383]	.368 [.361,.376]
80	40	40	1.75	.330 [.322,.338]	.282 [.275,.289]	.245 [.238,.252]	.246 [.239,.253]	.247 [.240,.254]
120	60	60	1.75	.463 [.455,.471]	.408 [.401,.416]	.353 [.346,.361]	.360 [.353,.368]	.355 [.347,.362]
160	80	80	1.75	.575 [.567,.583]	.522 [.514,.530]	.455 [.447,.463]	.464 [.456,.472]	.457 [.449,.465]
200	100	100	1.75	.655 [.648,.663]	.606 [.598,.614]	.533 [.525,.541]	.549 [.541,.557]	.533 [.525,.541]
240	120	120	1.75	.728 [.721,.735]	.686 [.679,.693]	.609 [.601,.617]	.623 [.615,.631]	.609 [.601,.617]
80	40	40	2.00	.466 [.458,.474]	.414 [.406,.422]	.362 [.354,.370]	.368 [.360,.376]	.366 [.358,.374]
120	60	60	2.00	.622 [.614,.630]	.569 [.561,.577]	.504 [.496,.512]	.515 [.507,.523]	.505 [.497,.513]
160	80	80	2.00	.739 [.732,.746]	.695 [.687,.702]	.626 [.618,.634]	.639 [.632,.647]	.628 [.621,.636]
200	100	100	2.00	.828 [.822,.834]	.794 [.787,.800]	.721 [.714,.728]	.732 [.725,.739]	.721 [.713,.728]
240	120	120	2.00	.886 [.881,.891]	.860 [.855,.866]	.793 [.787,.800]	.806 [.799,.812]	.796 [.789,.802]
80	40	40	2.25	.585 [.577,.592]	.534 [.526,.542]	.470 [.462,.478]	.473 [.465,.481]	.474 [.466,.482]
120	60	60	2.25	.749 [.742,.756]	.700 [.693,.708]	.634 [.626,.642]	.644 [.636,.652]	.637 [.629,.644]
160	80	80	2.25	.856 [.851,.862]	.825 [.819,.831]	.760 [.753,.767]	.767 [.760,.774]	.761 [.754,.768]
200	100	100	2.25	.924 [.920,.928]	.903 [.898,.908]	.854 [.848,.859]	.861 [.855,.866]	.855 [.849,.861]
240	120	120	2.25	.960 [.956,.963]	.946 [.942,.950]	.910 [.905,.914]	.916 [.911,.920]	.912 [.907,.917]
80	40	40	2.50	.677 [.669,.684]	.629 [.621,.637]	.561 [.553,.569]	.563 [.555,.571]	.563 [.555,.571]
120	60	60	2.50	.841 [.835,.846]	.805 [.799,.812]	.740 [.733,.747]	.751 [.744,.758]	.743 [.736,.750]
160	80	80	2.50	.924 [.919,.928]	.903 [.898,.907]	.855 [.849,.861]	.863 [.858,.869]	.857 [.851,.862]
200	100	100	2.50	.965 [.962,.968]	.954 [.950,.957]	.924 [.920,.928]	.929 [.925,.933]	.925 [.921,.929]
240	120	120	2.50	.987 [.985,.989]	.982 [.979,.984]	.961 [.958,.964]	.965 [.962,.968]	.962 [.959,.965]
80	40	40	2.75	.760 [.753,.767]	.715 [.707,.722]	.654 [.647,.662]	.655 [.647,.663]	.655 [.648,.663]
120	60	60	2.75	.900 [.896,.905]	.877 [.872,.882]	.826 [.820,.832]	.830 [.824,.836]	.827 [.821,.833]
160	80	80	2.75	.962 [.959,.965]	.950 [.947,.954]	.915 [.910,.919]	.921 [.916,.925]	.916 [.911,.920]
200	100	100	2.75	.985 [.983,.987]	.980 [.978,.982]	.962 [.958,.965]	.965 [.962,.968]	.962 [.959,.965]
240	120	120	2.75	.995 [.994,.996]	.993 [.992,.995]	.982 [.980,.984]	.984 [.982,.986]	.983 [.981,.985]
80	40	40	3.00	.816 [.810,.822]	.777 [.770,.784]	.726 [.719,.734]	.726 [.718,.733]	.727 [.720,.734]
120	60	60	3.00	.939 [.935,.943]	.921 [.916,.925]	.882 [.877,.887]	.886 [.881,.891]	.883 [.878,.888]
160	80	80	3.00	.980 [.978,.982]	.974 [.971,.976]	.954 [.950,.957]	.957 [.954,.960]	.955 [.951,.958]
200	100	100	3.00	.995 [.994,.996]	.993 [.992,.994]	.983 [.980,.985]	.984 [.982,.986]	.983 [.980,.985]
240	120	120	3.00	.999 [.998,.999]	.998 [.997,.999]	.994 [.993,.995]	.995 [.994,.996]	.994 [.993,.995]

Point estimates of the statistical power and 95% Wilson confidence intervals are reported

When the treatment had no effect (*β*_trt_ = 0), the nominal level of 0.05 was well preserved by almost all trial settings. However, we noted that the asymptotic ePolr test for all three models M1–3 was relatively liberal. In other words, more than 5% of the two-sided null hypotheses were rejected. This is due to the inadequately asymptotic normality approximation of the maximum likelihood estimator for small sample sizes. The model-based permutated ePolr test addressed exactly this challenge and successfully detected the expected effects within the simulation study, especially in trials with small sample sizes. Hence, the nominal level of 0.05 was well maintained by the permutated ePolr test. For the reporting and evaluation of the results, we therefore concentrated in particular on the comparison of the permutated ePolr test with the *t*-test, the Wilcoxon sum rank test, and ANCOVA.

In the following paragraphs, we will point out the most interesting findings based on the common goal of clinical trials to reach a statistical power of 80%. For the detailed simulation study results, see Tables 1, 2, and 3.

#### Upper extremity motor score (UEMS)

The application of the permutated ePolr test to model M1 clearly profited from the incorporation of the variable #seg and baseline-adjusted effect of the UEMS total sum score measured at time very acute. Accordingly, the estimated power of the permutated ePolr test was consistently higher than the estimated power of the *t*-test, Wilcoxon rank sum test, and ANCOVA. Thus, the increased power can be explained by the incorporation of baseline information and level of injury as strata in ePolr models. Given the postulated treatment effect exp(*β*_trt_) = 2.25, the targeted statistical power of 80% for the permutated ePolr test was reached in the trial setup where at least *N*≥160 trial participants are recruited. For the three conventional approaches, the same setup requires at least *N*≥240 trial participants to reach the targeted statistical power.

#### Spinal cord Independence measure (SCIM)

Models M2 and M3 also profited from the baseline-adjusted method. The estimated power also increased for the stratification based model M2 with SCIM total sum score and model M3 SCIM self-care subscore measurements. For model M2, the statistical power of 80% was first reached for the permutated ePolr test in the trial setup where the postulated treatment effect was exp(*β*_trt_) = 2 and where there were at least *N*≥200 recruited trial participants.

The analyses based on model M3 profited least from the new permutated ePolr test compared to the application with models M1 and M2. However, the statistical power of the simulation study in Table 3, still was slightly higher than the power of conventional analysis methods.

### Alternative analysis of the sygen® trial

We reanalyzed the Sygen® trial by testing for treatment effect on the UEMS total sum score. We applied all five approaches of the above-mentioned simulation study. None of the five approaches yielded significant results at the nominal level of 5%. The detailed results of the five analysis approaches are reported here. For the asymptotic and permutated Polr tests, we also report the results from additional stratification by study center.

- **t-test:** No significant difference in the estimated mean change control = 11.241 and treatment = 11.740 of the UEMS between trial arms, t(333) = 0.439, *p*-value = 0.661.
- **Wilcoxon rank sum test:** No significant difference between trial arms, W = 13816, *p*-value = 0.812.
- **ANCOVA:** No significant difference between trial arms adjusted for baseline score measurements. F-statistic: 190.8, *p*-value = 0.651
- **Asymptotic ePolr test:** No significant shift in motor score probabilities associated with trial arm stratified by UEMS baseline measurements and number of segments below motor level (#seg), exp(*β*_trt_) = 1.142 (95%-CI: [ 0.778,1.678], odds ratio of treatment versus control group), *p*-value = 0.234. Additional stratification by study center leads to exp(*β*_trt_) = 1.186 (95%-CI: [ 0.797,1.765]), *p*-value = 0.399.
- **Permutated ePolr test:** No significant difference in score contributions of UEMS measurements at time acute III and trial arm stratified by UEMS baseline measurements, Z = −0.655, *p*-value = 0.5237. Here, additional stratification by study center results in Z = −0.890, *p*-value = 0.3677.

These results can by no means be generalized owing to the strongly selected subsample of patients considered, the different outcomes analyzed, and the different scopes of analysis. Nonetheless, the estimated ORs from the enhanced proportional odds models showed a tendency towards positive effect of treatment on the UEMS, which means that treated patients had on average a slightly better recovery than patients in the treatment-naive group. Especially for the baseline-adjusted proportional odds model (asymptotic ePolr test), the result implies that the odds of a participant in the treatment group of achieving up to a given motor score were exp(*β*_trt_) = 1.142 times the odds of a participant with similar characteristics in the control arm. This indicates a slightly better recovery of treated patients with a *p*-value considerably smaller than the *p*-values obtained from the *t*- or Wilcoxon-tests, and ANCOVA. Nevertheless, we still believe that generalizations of our results to the overall validity of the Sygen^®^ trial and its compound cannot be drawn.

## Discussion

### Randomized clinical trial simulation

As outlined above, conventional approaches of testing for treatment effects in two-armed randomized clinical trials with ordinal sum score outcomes face some limitations. Our novel method based on the baseline-adjusted proportional odds model estimates a global treatment effect conditioning on the total sum score, while allowing for baseline adjustment and confidence interval-based inference. We exemplified the method by applying the models to ordinal outcomes of a future neurological clinical trial. However, the model setup is generalizable to any type of ordinal response measure with a considerably high number of non-reducible outcome categories.

A direct comparison of our proposed method and the routinely employed analysis methods based on statistical power showed that the permutated ePolr test has higher statistical power in every postulated trial setting. However, the differences in statistical power of the model-based methods and conventional approaches are not as large for the SCIM total sum score model M2 and for the SCIM self-care subscore model M3 as for the UEMS total sum score model M1. Based on discussions with neurologists, this is likely explained by the influence of other factors, such as concomitant damage to other body tissues, inflammation or circulator disturbances. These factors can diminish the performance of SCIM activity items, especially at very acute assessment times. Hence, we conclude that in terms of neurological recovery, a baseline adjustment is more advantageous for the analysis of the UEMS total sum score than for the SCIM total sum score and self-care subscore.

Although the asymptotic ePolr test might be relatively liberal, in a clinical setting, it has the clear advantage of confidence interval-based inference. This significance test not only gives a *p*-value for the randomized clinical trial group comparison, but also quantifies the difference and provides a model-based confidence interval.

It was recently pointed out [41] that the Wilcoxon rank sum test can be understood as the permutation score test in a Polr model. Consequently, the Wilcoxon test is particularly powerful against OR alternatives. In contrast to the *t*-test, which is powerful against shift alternatives, the Wilcoxon rank sum test implicitly focuses on the same OR parameter exp(*β*_trt_) that our models M1–3 are built upon. We can thus conclude that the superior power demonstrated in the simulation experiments arises from the incorporation of baseline information by stratification and not from different parametrizations or distributional assumptions. This conclusion is also supported by [42], stating that stratum variables should be considered in design and analysis when outcome categories are heavily dependent on membership of some stratum.

Instead of comparing outcome scores obtained at a specific time point, the score trajectory over time could be modelled, with a treatment effect parameter distinguishing between the two groups. The procedure described by Parsons [13] directly targets such a situation by applying a marginal proportional odds models for repeated measurements where the baseline log-odds function *h* is parameterised by orthogonal polynomials and parameter inference relies on GEEs. An alternative way of approaching this problem could be motivated by marginally interpretable transformation models for longitudinal observations [43], where stratum-specific baseline log-odds functions *h* could be parameterised as described here. The latter approach allows for simple maximum-likelihood inference and a head-to-head comparison of both approaches remains an interesting topic for future research.

### Alternative analysis of the sygen® trial

The alternative analysis of a subsample of patients from the Sygen® trial underlined the straightforward implementation of our method. For this specific example, our model-based method enhanced the conventional analysis approaches of UEMS total sum scores by allowing for stratification based on the UEMS total sum score at baseline as well as baseline adjustment by #seg in terms of the patient’s individual motor level. The confidence interval-based inference as part of the reported results of the asymptotic ePolr test is a clear advantage of the new method. We no longer only report a *p*-value as a result of the significance test but also a confidence interval of the quantified global treatment effect *β*_trt_. The treatment effect exp(*β*_trt_) directly estimated from the model has the clear clinical interpretation as an OR that compares the treatment group with the control group.

### Computational details

All computations were performed using R version 3.6.1 [44]. The tramR add-on package [28] was used to estimate the ePolr models. The underlying statistical theory is described in [24]. A blueprint for the estimation of the conditional distribution functions of the UEMS at time acute III (UEMS3) for the treatment group and control group is shown below. Data are available in a data frame SCI_OR_3 with variables UEMS at time very acute (UEMS0, numeric) and acute III (UEMS3, numeric), #seg (factor), and trtmt (binary: 0 for control group and 1 for treatment group). The factor variable #seg is defined as the number of left and right spinal segments below motor level that are at or more caudal than C5 and at or more rostral than T1 in three strata ([0,6],[7,8],[9,10]).


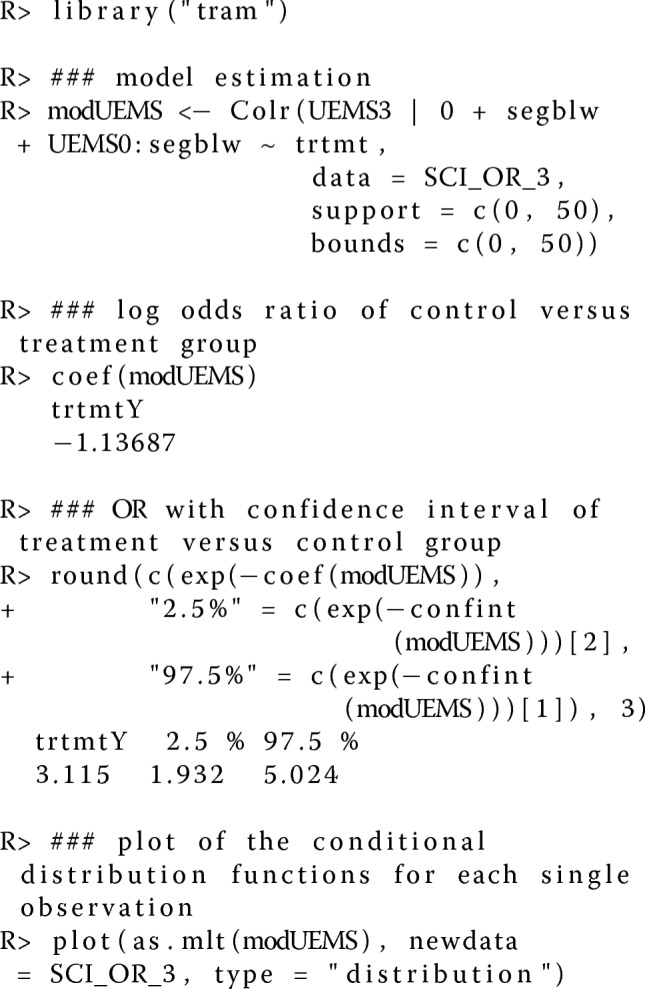


The treatment effect *β*_trt_ estimated with the readily implemented Colr() function of the R add-on package tram compares the control group with the treatment group on the scale of the log-OR. Hence, large negative values of the linear predictor *β*_trt_ correspond to large expected total sum scores and thus high treatment effect. The inversion of the exponential of the treatment effect exp(*β*_trt_) results in the conventional measure of association that compares the treatment group with the control group on the OR scale. R code implementating the simulation study is available online (doi: http://dx.doi.org/10.5281/zenodo.1411339).

## Conclusion

We introduced baseline-adjusted proportional odds models, which can be considered as extensions of the well-known proportional odds model to estimate the treatment effect of ordinal outcomes from a clinical trial. Our proposed method extends the conventional analysis approaches by stratifying based on the specific total sum score measurements at trial baseline and potentially additional baseline variables. Further extensions of our method can be tailored to individual trial designs, which leads to improved analyses of complex trial designs.

The proposed models result in a global treatment effect measure that can be directly interpreted on the original ordinal scale of the outcome measures. Hence, the clear interpretation of the global treatment effect, the superior statistical power compared to that of conventional analysis approaches, as well as the open-source availability for the estimation of such models are strong arguments for the use of such methods for the analysis of future clinical trials.

## Data Availability

The data sets supporting the conclusions of this article are not publicly available. Interested researcher can apply for data access to the responsible organization; access is usually granted only for research purposes. The R code implementing the simulation study is available online (doi: http://dx.doi.org/10.5281/zenodo.1411339).

## Abbreviations

ANCOVA: Analysis of covariance
EMSCI: European multicenter study about spinal cord injury
ePolr: Enhanced proportional odds logistic regression
GEE: Generalised estimating equations
GRM: Graded response model
ISNCSCI: International standards for neurological classification of spinal cord injury
NISCI: Nogo inhibition in spinal cord injury trial
OR: Odds ratio
PCM: Partial credit model
Polr: Proportional odds logistic regression
RCT: Randomized clinical trial
SCI: Spinal cord injury
SCIM: Spinal cord independece measure
SCIMsc: Spinal cord independece measure self-care
UEMS: Upper extremity motor scores
