# Correction to: Baseline-adjusted proportional odds models for the quantification of treatment effects in trials with ordinal sum score outcomes

**DOI:** 10.1186/s12874-020-01028-5

**Published:** 2020-07-09

**Authors:** Muriel Buri, Armin Curt, John Steeves, Torsten Hothorn

**Affiliations:** 1 Department of Biostatistics, Epidemiology, Biostatistics and Prevention Institute, University of Zurich, Hirschengraben 84, CH-8001 Zurich, Switzerland; 2 University Hospital Balgrist, Spinal Cord Injury Center, Forchstrasse 340, CH-8008 Zurich, Switzerland; 3 International Collaboration On Repair Discoveries (ICORD), University of British Columbia, Vancouver/Kelowna, Canada

**Correction to: BMC Med Res Methodol (2020) 20:104**

**https://doi.org/10.1186/s12874-020-00984-2**

Following publication of the original article [1], the authors would like to correct the formula of the Polr model equation in the paragraph under the heading **Enhanced proportional odds logistic regression (ePolr)**.

The formula currently reads:

$$ \mathrm{P}\left({\mathrm{Y}}^{\mathrm{III}}\le {\mathrm{y}}^{\mathrm{III}}\left|x\right.\;\right)=\mathrm{expit}\left(h\left({\mathrm{y}}^{\mathrm{III}}\left|{\mathrm{y}}^0\right.\right)+\beta \cdot x\right)\;\left(\mathrm{Polr}\right) $$

The formula should read:

$$ \mathrm{P}\left({\mathrm{Y}}^{\mathrm{III}}\le {\mathrm{y}}^{\mathrm{III}}\left|x\right.\;\right)=\mathrm{expit}\left(h\left({\mathrm{y}}^{\mathrm{III}}\right)+\beta \cdot x\right)\;\left(\mathrm{Polr}\right) $$

The original article [1] has been corrected.
